# Supplementary material for: Dynamics of asymmetric membranes and interleaflet coupling as intermediates in membrane fusion
Source: Biophys J. 2022 Oct 5;122(11):1985–95. doi: 10.1016/j.bpj.2022.10.006 (PMC10257014; doi:10.1016/j.bpj.2022.10.006)
Supplement: Document S1. Figures S1–S5 and Tables S1 and S2 [file mmc1.pdf]

Biophysical Journal, Volume *122*

Supplemental information

Dynamics of asymmetric membranes and interleaflet coupling as intermediates in membrane fusion

Marcos Arribas Perez and Paul A. Beales

Supplemental figures:

Figure S1

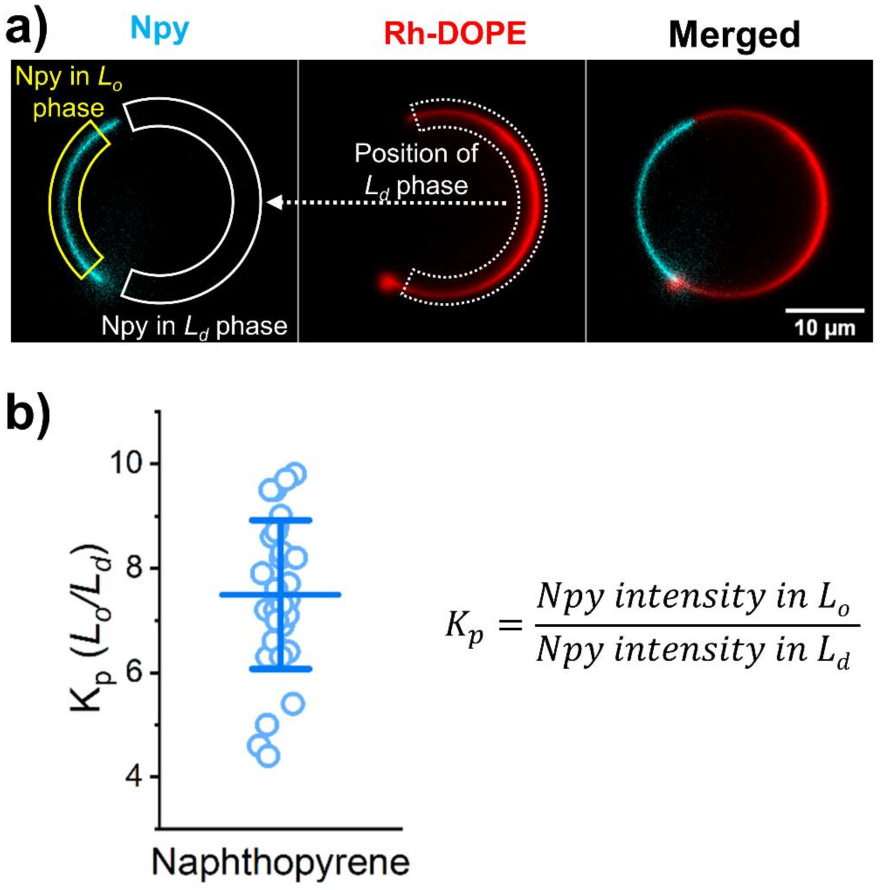

Partition coefficient (K_p) of naphthopyrene in phase separated GUVs. a) Confocal microscopy image of a phase separated GUV after fusion. Napthopyrene (Npy) is shown in cyan and Rhodamine-DOPE (Rj-DOPE) in red. To calculate the K_p of Naphthopyrene, we first measured the average fluorescence intensity of Npy over the ordered phase (yellow ROI) and the disordered phase (white ROI). The region equivalent to the disordered phase was selected in the Rh-DOPE channel (dashed white ROI). B) K_p values obtained from the analysis of 32 GUVs (open circles in plot). The K_p values were calculated using the formula in the figure by dividing the Npy fluorescence intensity in L_o phase over the Npy fluorescence intensity in the L_d phase. We obtained an average K_p of 7.5 ± 1.4 (mean ± SD). Fluorescence intensity values and K_p of individual GUVs are shown in Table S1.

Figure S2.

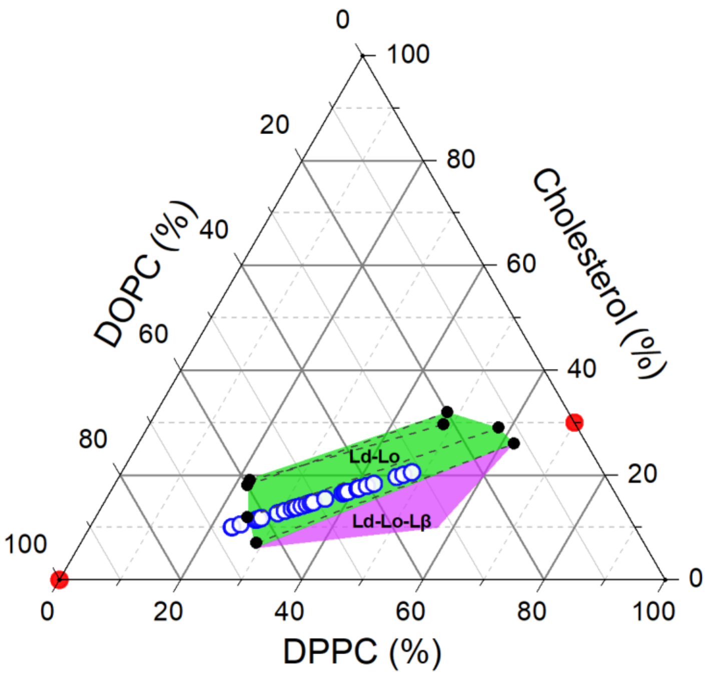

Representation of the lipid composition of the final of fused GUV calculated from the relative surface area of the initial GUVs. Red circles indicate the lipid composition of the initial L_d (100% DOPC) and L_o GUVs (DPPC:Chol 70%:30%). Open blue circles represent the compositions calculated in this work as shown in Table S2. Dashed black lines and black dots show the NMR tie-lines and end points of at the two-phase L_d-L_o region (shaded in green) of DOPC:DPPC:cholesterol membranes at 20°C shown in a previous study by Veatch *et al.* (1). L_d-L_o-S_o three-phase region reported in (1) is shown in magenta.

Figure S3.

a)

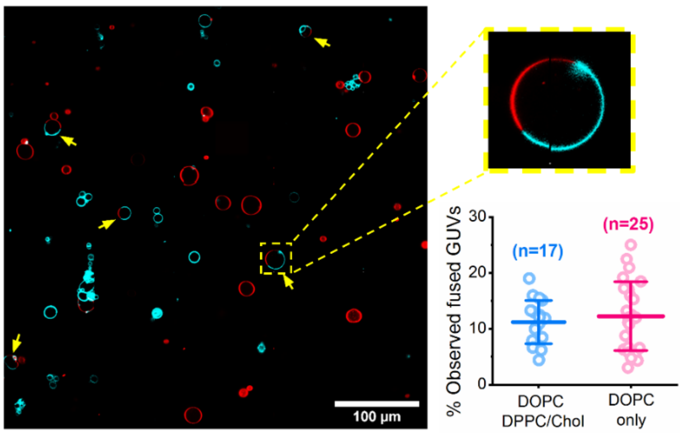

b)

GUVs composition	Total fusion events recorded	Direct full fusion	Hemifusion-fusion	Gentle merging
DOPC only	20	35% (n=7)	30% (n=6)	35% (n=7)
DOPC + DPPC:Chol (7:3)	32	53% (n=17)	47% (n=15)	0% (n=0)

a) Confocal microscopy tile scan after incubating DOPC GUVs and DPPC:Cholesterol (7:3) GUVs in a 1:1 volume ratio with 25 µg/ml SiO_2 NPs. Yellow arrows highlight the phase separated GUVs. Top left image shows a zoomed in phase separated GUV. Plot shows the distribution of the percentage of fused GUVs observed from tile scans in samples of DOPC GUVs mixed with DPPC:Chol (7:3) GUVs in a 1:1 volume ratio (blue) and in control samples of DOPC GUVs (magenta) where half of the GUVs are labelled with Rh-DOPE and the other half with DiO. Data are presented as mean ± SD, circles indicate the percentage of fused GUVs observed in each tile scan (number of tile scan images analysed indicated in the plot). In samples of DOPC and DPPC:Chol GUVs the proportion of observed fused (phase separated) GUVs was 11.3 ± 3.9 % and in samples of only DOPC GUVs the proportion of observed fused GUVs was 12.5 ± 5.9 %. The proportion of observed fused GUVs in the two conditions does not show statistical difference (one-way ANOVA with a $p < 0.5$ post-hoc Bonferroni test). Over 1000 individual GUVs were counted for each condition. **b)** Table shows the percentage of recorded fusion events corresponding to each fusion pathway described in our previous study (direct full fusion, hemifusion-fusion and gentle membrane merging) (2) when both GUVs fusion are composed of only DOPC and when one is DOPC and the other DPPC:Chol (7:3). While the percentage of the 3 pathways is very similar in DOPC-DOPC GUV fusion, the gentle membrane merging pathway is suppressed when one of the vesicles undergoing fusion is a liquid ordered DPPC:Chol GUV.

Figure S4.

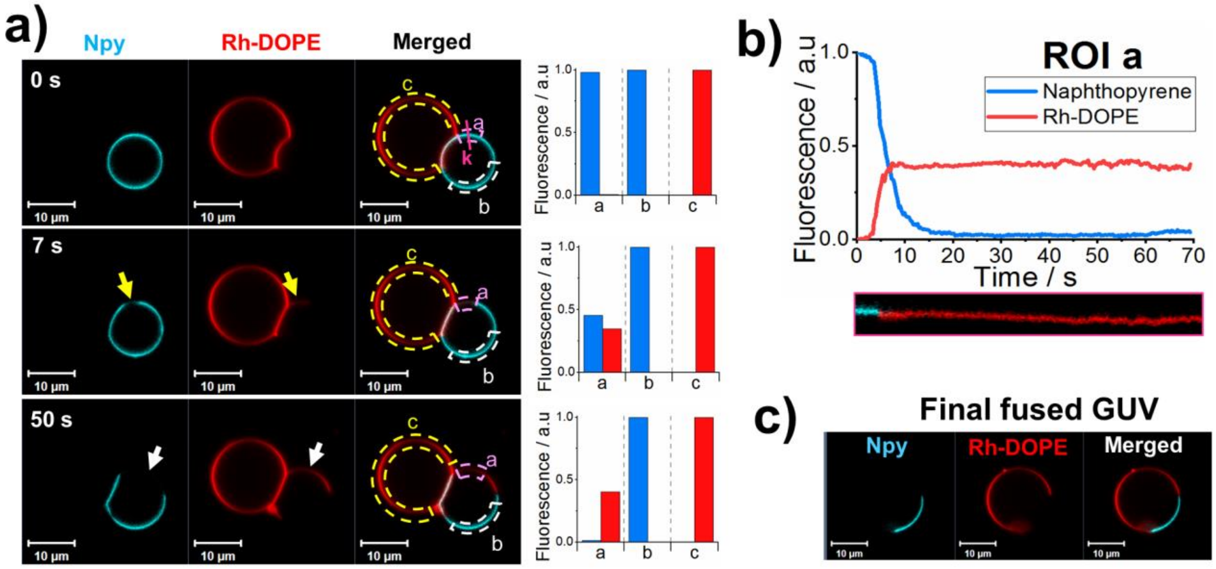

Asymmetric membrane domains and interleaflet coupling in hemifused GUVs. a) Confocal microscopy time-lapse images of GUVs during hemifusion. Naphthopyrene (Npy) (cyan) and Rh-DOPE (red) are used as L_o and L_d markers, respectively. Yellow arrows indicate regions of the membrane where both dyes are colocalised (asymmetric domains) and white arrows indicate regions of the membrane where the L_o marker has been excluded from the membrane due to interleaflet coupling. Bar plots show the normalised fluorescence intensity measured in the ROIs "a","b" and "c" shown in the merged channel. Fluorescence intensity in "ROI b" and "ROI c" corresponds to the maximum intensity of Npy and Rh-DOPE, respectively, and the background is used as minimum intensity. "ROI a" shows an initial rise in Rh-DOPE intensity simultaneous to a reduction of Npy fluorescence to about half compared to its maximum (7 s micrograph). Micrographs at 50 s show a complete exclusion of Npy from that region of the membrane while the intensity of Rh-DOPE in "ROI a" remains close to 0.5. b) Normalised fluorescence intensity of Rh-DOPE (red line) and Npy (blue line) in "ROI a" against time. Kymograph shows fluorescence signal of the dyes over time at the pink line indicated as "k" in the 0 s micrographs in a). c) Phase separated GUV after full fusion.

Figure S5.

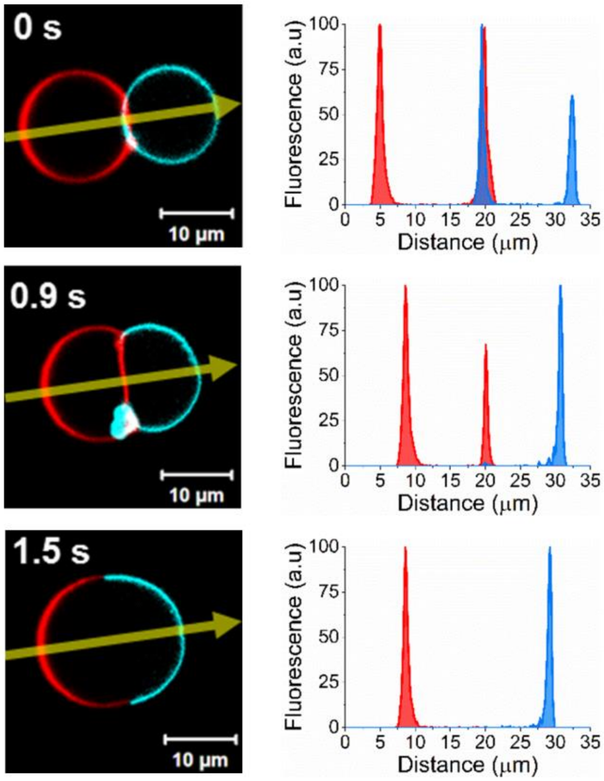

Confocal microscopy micrographs showing Naphthopyrene (Npy) exclusion from the contact region before fusion. Plots show the fluorescence intensity profile of Npy and Rh-DOPE across the yellow arrow indicated in the micrographs.

Supplemental tables:

Table S1. Fluorescence intensity of Naphthopyrene dye in L_d and L_o domains and K_p values of individual phase separated GUVs.

	Npy intensity (a.u.)		
	L_o	L_d	K_p
	32.2	5.1	6.3
	27.5	2.9	9.5
	68.7	7.2	9.5
	39.3	4	9.8
	33.1	4.2	7.9
	26.2	3.8	6.9
	29.2	3.3	8.8
	70.8	9.8	7.2
	36.8	4.1	9
	23.1	3.6	6.4
	47.8	9.6	5
	39.3	8.6	4.6
	26.1	2.7	9.7
	34.6	4.7	7.4
	34.3	4	8.6
	50.2	6.1	8.2
	30.8	3.7	8.3
	41.1	5.5	7.5
	45.7	6.4	7.1
	36.2	5	7.2
	34.7	4	8.7
	35.8	4.9	7.3
	49.8	6.5	7.7
	48.2	5.9	8.2
	39.1	5.6	7
	40.9	9.2	4.4
	31.3	4.1	7.6
	33.2	5	6.6
	36.2	6.7	5.4
	33.2	3.8	8.7
	45.7	6.5	7
	26.6	4.2	6.3
**Mean**	38.4	5.3	7.5
**SD**	10.8	1.9	1.4

Table S2. Calculation of lipid composition in the fused phase separated GUVs from the relative surface area of the initial GUVs.

Diameter (μm)		Radius (μm)		Surface area (μm²)		Surface area fraction		Lipid % in fused GUV *		
L_d GUV	L_o GUV	L_d GUV	L_o GUV	L_d GUV	L_o GUV	L_d GUV	L_o GUV	DOPC	DPPC	Chol
17.2	12.2	8.6	6.1	929	468	0.67	0.33	67	23	10
13.8	9.8	6.9	4.9	598	302	0.66	0.34	66	24	10
16.7	12.3	8.35	6.15	876	475	0.65	0.35	65	25	11
14	11	7	5.5	616	380	0.62	0.38	62	27	11
14.6	11.5	7.3	5.75	670	415	0.62	0.38	62	27	11
15.1	12	7.55	6	716	452	0.61	0.39	61	27	12
13.2	10.6	6.6	5.3	547	353	0.61	0.39	61	27	12
14.8	12.7	7.4	6.35	688	507	0.58	0.42	58	29	13
14.4	12.7	7.2	6.35	651	507	0.57	0.43	57	30	13
10.7	9.7	5.35	4.85	360	296	0.56	0.44	56	31	13
14.9	13.7	7.45	6.85	697	590	0.55	0.45	55	32	14
15.9	14.7	7.95	7.35	794	679	0.55	0.45	55	32	14
10.4	9.8	5.2	4.9	340	302	0.54	0.46	54	32	14
18.5	17.8	9.25	8.9	1075	995	0.54	0.46	54	32	14
17.2	16.8	8.6	8.4	929	887	0.53	0.47	53	33	14
14.7	14.5	7.35	7.25	679	661	0.52	0.48	52	34	14
12.2	12.6	6.1	6.3	468	499	0.51	0.49	51	34	15
13.7	15.1	6.85	7.55	590	716	0.51	0.49	51	34	15
11.2	12.5	5.6	6.25	394	491	0.48	0.52	48	36	16
15.4	17.2	7.7	8.6	745	929	0.45	0.55	45	39	17
10.1	11.4	5.05	5.7	320	408	0.45	0.55	45	39	17
12.2	14.3	6.1	7.15	468	642	0.44	0.56	44	39	17
12.6	14.8	6.3	7.4	499	688	0.44	0.56	44	39	17
13.1	15.4	6.55	7.7	539	745	0.42	0.58	42	41	17
14.4	17	7.2	8.5	651	908	0.42	0.58	42	41	17
18.9	23	9.45	11.5	1122	1662	0.42	0.58	42	41	17
12.3	15.4	6.15	7.7	475	745	0.42	0.58	42	41	17
8.5	11.7	4.25	5.85	227	430	0.4	0.6	40	42	18
11.5	14.3	5.75	7.15	415	642	0.39	0.61	39	43	18
9.7	13.4	4.85	6.7	296	564	0.39	0.61	39	43	18
15.1	13.6	7.55	6.8	716	581	0.35	0.65	35	46	20
16.4	14.1	8.2	7.05	845	625	0.34	0.66	34	46	20
Hemifusion intermediate state observed					**Mean**	**0.51**	**0.49**	**50.7**	**34.5**	**14.8**
Direct full fusion					**SD**	**0.1**	**0.1**	**9.2**	**6.5**	**2.8**

* The lipid composition was calculated by multiplying the surface area fraction of the appropriate GUV by the mol% of that lipid in the initial GUV (L_d GUV in the case of DOPC and L_o GUV for DPPC and cholesterol):

$$\%\ \text{DOPC in fused GUV} = \text{ Initial DOPC \% in } L_d \text{ GUV (100\%)x } L_d \text{ surface area fraction}$$

$$\%\ \text{DPPC in fused GUV} = \text{ Initial DPPC \% in } L_o \text{ GUV (70\%)x } L_o \text{ surface area fraction}$$

$$\%\ \text{Cholesterol in fused GUV} = \text{ Initial Cholesterol \% in } L_o \text{ GUV (30\%)x } L_o \text{ surface area fraction}$$

References

1. Veatch, S. L., O. Soubias, S. L. Keller, and K. Gawrisch. 2007. Critical fluctuations in domain-forming lipid mixtures. Proceedings of the National Academy of Sciences. 104(45):17650-17655, doi: doi:10.1073/pnas.0703513104
2. Arribas Perez, M., and P. A. Beales. 2021. Biomimetic Curvature and Tension-Driven Membrane Fusion Induced by Silica Nanoparticles. *Langmuir*. 37(47):13917-13931, doi: 10.1021/acs.langmuir.1c02492
